# Core and Valence Level Photoelectron Spectroscopy of Nanosolvated KCl

**DOI:** 10.1021/acs.jpca.1c01539

**Published:** 2021-05-26

**Authors:** Eetu Pelimanni, Lauri Hautala, Andreas Hans, Antti Kivimäki, Mati Kook, Catmarna Küstner-Wetekam, Lutz Marder, Minna Patanen, Marko Huttula

**Affiliations:** † Nano and Molecular Systems Research Unit, Faculty of Science, University of Oulu, P.O. Box 3000, FI-90014 Oulu, Finland; ‡ Universität Kassel, Institut für Physik und CINSaT, Heinrich-Plett-Straße 40, 34132 Kassel, Germany; § MAX IV Laboratory, Lund University, P.O. Box 118, SE-22100 Lund, Sweden; ∥ Institute of Physics, University of Tartu, W. Ostwaldi 1, EE-50411 Tartu, Estonia

## Abstract

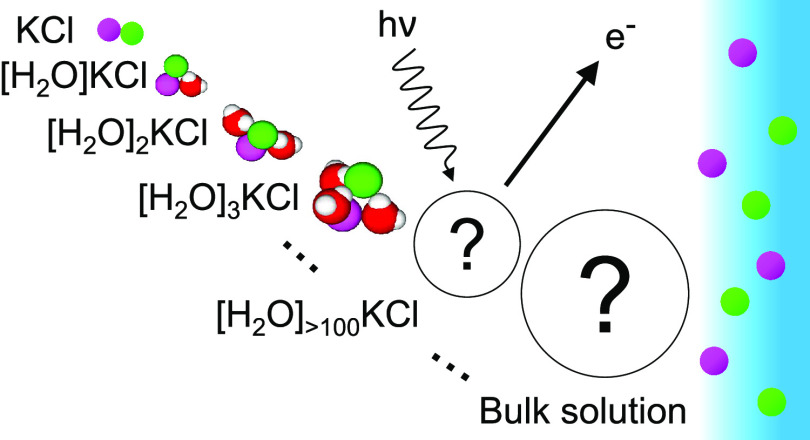

The solvation of alkali and halide ions in the aqueous environment has been a subject of intense experimental and theoretical research with multidisciplinary interests; yet, a comprehensive molecular-level understanding has still not been obtained. In recent years, electron spectroscopy has been increasingly applied to study the electronic and structural properties of aqueous ions with implications, especially in atmospheric chemistry. In this work, we report core and valence level (Cl 2p, Cl 3p, and K 3p) photoelectron spectra of the common alkali halide, KCl, doped in gas-phase water clusters in the size range of a few hundred water molecules. The results indicate that the electronic structure of these nanosolutions shows a distinct character from that observed at the liquid–vapor interface in liquid microjets and ambient pressure setups. Insights are provided into the unique solvation properties of ions in a nanoaqueous environment, emerging properties of bulk electrolyte solutions with growing cluster size, and sensitivity of the electronic structure to varying solvation configurations.

## Introduction

Aqueous alkali and halide ions are involved in a myriad of natural and technological processes, yet their structural and electronic properties are not fully understood. Clusters play a central role in probing the detailed interplay of ions with water molecules, in both experiments and theory.^1−6^ Clusters can be considered as simplified models of bulk solutions, but they are also in many ways unique as the solvation structures and dynamics are subject to, e.g., the high surface-to-volume ratio and distinct thermodynamic properties. There is currently much interest in the solvation properties of ions in finite dimensions, such as saline atmospheric particles and nanochannels.^7−9^

The supersonic expansion + pickup principle has proved efficient for the production of mixed water clusters with dopant species and their subsequent characterization in the gas phase.^10^ It has recently been applied to study alkali halides in water clusters^11,12^ as well as anhydrous alkali halide clusters^12−15^ with X-ray photoelectron spectroscopy (XPS). It was reported that the binding energies (BEs) of nanosolvated alkali and halide ions are insensitive to the counterions in water clusters with a few hundred water molecules,^11^ similar to what has been observed in bulk solutions.^16^ Notable BE discrepancies however remained, especially for the anion core levels that were attributed to calibration uncertainty in bulk level studies. In a later study, the effect of changing salt concentration was addressed with signatures of ion pairing observed in the core level spectra.^12^

A particular question lies on what size regime and how well the large cluster size limit corresponds to properties of bulk solutions.^16,17^ Winter et al.^16^ considered that the convergence of ionization potentials to bulk values is relatively slow due to long-range solvent polarization effects, the population of interior and surface solvation sites may differ, and structural properties of low-temperature clusters differ from bulk solutions. By performing XPS on salt-containing clusters of growing size, the sensitivity of the electronic structure to varying solvation configurations and the extent of ion–ion and ion–solvent interactions can be investigated. These questions are particularly timely now that electron spectroscopy is being increasingly applied to in situ and operando chemical analysis in wet conditions, largely enabled by liquid microjets^18^ and ambient pressure setups,^19^ which are available also at modern high brilliance synchrotrons.^20^ In particular, surface-sensitive XPS has gained popularity in studying the liquid–vapor interface with atmospheric implications,^21−23^ and the role of finite size effects should be investigated.

In this work, we report core and valence level (Cl 2p, Cl 3p, K 3p) photoelectron spectra of KCl embedded in water clusters with hundreds of water molecules. The sensitivity of the electronic structure to cluster size and salt concentration is assessed, and the intrinsic properties of the clusters are discussed. KCl is a naturally abundant alkali halide with various applications, and its electronic structure has been broadly investigated with XPS as free molecules,^11,14,24^ clusters of [H_2_O]*_N_*[KCl]*_k_*^11^ and [KCl]*_N_*,^14^ bulk KCl solutions,^16,25^ and solid KCl.^24^ Unique solvation properties in the nanoaqueous environment can thus be assessed by a detailed comparison of the results to these various reference systems.

## Experimental Methods

**Figure 1:**
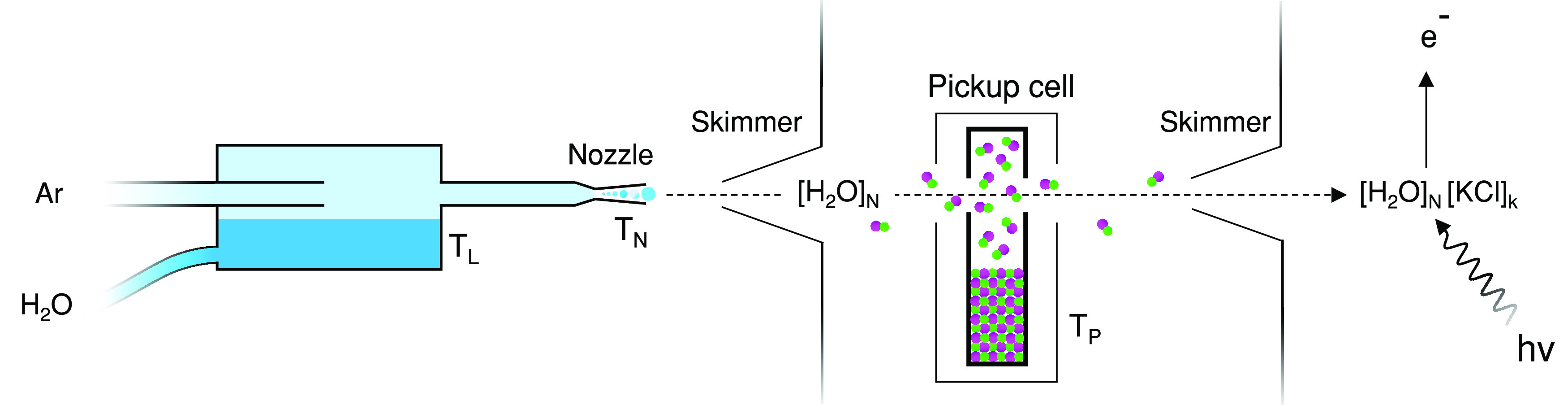
Schematic illustration of the experiment.

The experiment was carried out at the gas phase end station^26^ of the newly established FinEstBeAMS beamline^27^ at MAX IV synchrotron laboratory (Lund, Sweden). The clusters were generated with the new Molecular Multi Use Setup for Clusters Emission (MUSCLE), which is based on the earlier EXMEC^28^ design. Figure 1 shows a schematic illustration of the experiment. Pure water clusters were generated in a continuous supersonic gas expansion and subsequently doped with KCl. Ultrapure water (18.2 MΩ·cm at 25 °C) and anhydrous potassium chloride powder of ≥99% purity (Sigma-Aldrich) were used. A conical nozzle with a minimum diameter of *d*_N_ = 188 ± 5 μm, a half opening angle α of 5 ° and a ∼20 mm long expansion cone were used. The temperature of the nozzle was set to *T*_N_ = 100 ± 2 °C. Two different cluster size distributions are compared to assess the size sensitivity of the spectra. With water heated to *T*_L_ = 80 °C, the smaller size was generated at a *P* = 500 ± 10 mbar pure water vapor expansion pressure. The larger size was generated by using argon as a carrier gas with a total *P* = 1000 ± 100 mbar expansion pressure. The expansion pressure was monitored using a gas-independent absolute capacitance manometer. Employing the conventional scaling laws,^29,30^ the mean number of water molecules is estimated to be ⟨*N*⟩_S_ = 160_50_^250^ (*d* ∼ 2 nm^31^) and ⟨*N*⟩_L_ = 500_100_^1000^ (*d* ∼ 3 nm) for the smaller and larger sizes, respectively. The lower and upper indices indicate the minimum and maximum estimates of the mean size. The widths of the size distributions are expected to be ∼⟨*N*⟩.^29^ The liquid, the nozzle, and the pickup cell were Joule-heated, and the temperatures were monitored using K-type thermocouples. Beam skimmers were placed before (∼0.8 mm orifice) and after (∼3 mm orifice) the pickup cell. Details of the used pickup cell are given elsewhere.^32^

Photoelectron spectra were measured using a hemispherical deflection analyzer, Scienta R4000, aligned perpendicular to both the cluster beam and the X-ray beam. The observation angle was parallel to the light polarization axis. In all measurements, the analyzer was operated at 50 eV pass energy, and a 0.8 mm entrance slit was used. The K 3p and Cl 3p valence spectra were measured in the same scan with 100 eV photon energy and a total experimental resolution of ∼100 meV, estimated from the Gaussian full width at half-maximum (FWHM) of the Ar 3s photoelectron peak. The Cl 2p spectra were measured separately with a photon energy of 230 eV and a resolution of ∼300 meV, estimated from the Gaussian FWHM of the Ar 2p photoelectron peaks measured separately with 280 eV (lifetime broadening from ref (33)). The BE scale was calibrated to the 1b_1_ level of molecular water at 12.615 eV,^34^ thus finding good agreement also for the Ar 3p, 3s, and 2p lines with their literature values^33,35^ (and references therein). Details of the cluster source parameters and measured statistics for all of the presented spectra are included in the Supporting Information (SI), Table S1. Peak analysis was performed using Igor Pro software from WaveMetrics with a custom-made least-squares curve-fitting package SPANCF.^36,37^ Details of the peak-fitting procedures are likewise included in the Supporting Information.

## Results and Discussion

### Pure Water Clusters

When comparing clusters to bulk solutions, it should be considered that the electronic structure and solvation dynamics are subject to the temperature and structure of the water network.^38,39^ Properties of pure water clusters, without pickup, are therefore discussed first. The phase of water clusters depends on the cooling rate in the expansion, final temperature, and size.^31,40^ The melting point of water clusters is lower than that of bulk water, on the order of ∼170 K for *N* = 160 and ∼210 K for *N* = 500.^31^ The temperature of the clusters cannot be directly retrieved from the present data but typical estimates are well below or around these values.^40−44^ Water clusters of this size range are often presumed frozen and even referred to as “ice nanoparticles”.^44^ The water network of small water clusters is largely amorphous (as of liquid), and partial crystallinity can occur when *N* ≳ 90.^40^ Irregular geometries may also occur.^45^

To evaluate the similarity of the electronic structure of our pure water clusters to bulk water, we consider the vertical binding energy (VBE) of the water 1b_1_ valence level, which decreases with increasing cluster size and bridges the transition from single molecules to bulk.^30^ At present, there is however no widespread consensus on the precise 1b_1_ VBE of the bulk liquid water, which remains actively discussed due to its common use as a calibration reference of liquid jet experiments.^46−54^ Values have been reported at a broad energy range, from the early assignment of 11.16(4) eV by Winter et al.^46^ to the recent assignment of 11.67(15) eV by Perry et al.^49^ The uncertainty relates to the inherent charging of the liquid, which is often compensated with bias voltages and/or a specific electrolyte concentration.^52^ This was not necessary for the present experiment, making the calibration to gas-phase references more straightforward. Inconsistencies in VBEs for both the solvent and solute levels between the clusters in our experiment and bulk solutions can thus reflect both finite size effects and calibration differences, and the different contributions are difficult to distinguish as such. On the other hand, the remaining uncertainty encourages one to consider how well the bulk assignments are in line with the size-dependent and eventually converging trends of gas-phase clusters.

**Figure 2:**
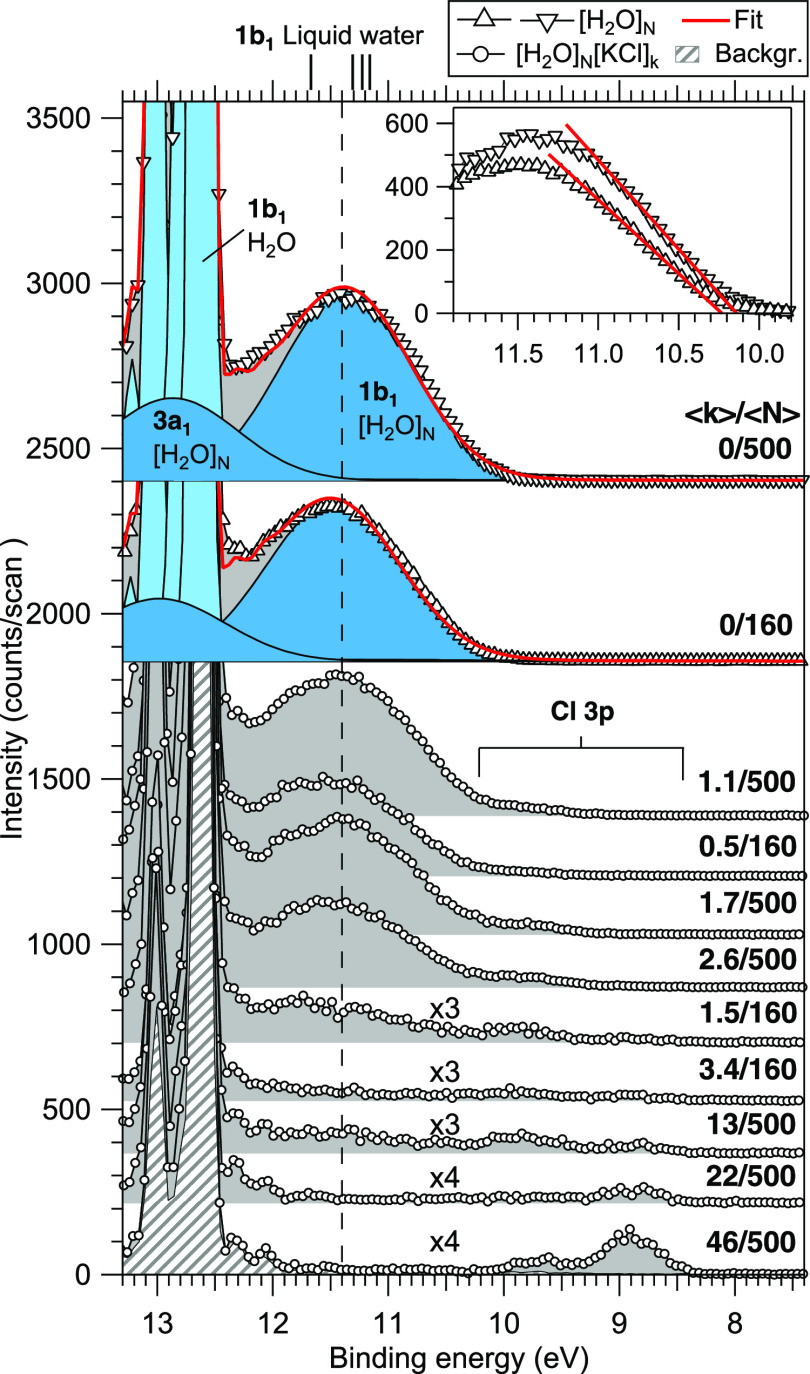
Photoelectron spectra in the water 1b_1_ valence region, measured with a photon energy of 100 eV. Vertical offsets have been applied for clarity. From top to bottom, the spectra are organized in order of increasing concentration, ⟨*k*⟩/⟨*N*⟩, where ⟨*k*⟩ denotes the (lower limit of) mean number of picked-up molecules and ⟨*N*⟩ the mean initial cluster size (160_50_^250^ or 500_100_^1000^). A background spectrum (scaled independently for better comparison) without clusters is shown for reference. VBE and ionization threshold (inset) fits are shown for the pure water cluster spectra. For comparison, VBEs of liquid water reported by others are given on top of the graph.^46−49^ The dashed line at 11.4 eV is a guide to the eye.

The 1b_1_ valence spectra obtained in the present experiment are shown in Figure 2. For both water clusters and liquid jets, the 1b_1_ VBE is commonly obtained from a single Gaussian fit, and the same procedure is thus adopted here. For the smaller and larger pure water clusters (without pickup), we obtain VBEs (threshold BEs) of 11.5 ± 0.1 eV (10.2 ± 0.1 eV) and 11.4 ± 0.1 eV (10.1 ± 0.1 eV), respectively. The error limits account for the experimental resolution and calibration accuracy, but lowering the VBE by 0.1 eV for the larger size is clearly resolved. The threshold energies were obtained from extrapolated line fits to the low BE side, as shown in the inset on the top right corner of Figure 2. With a similar treatment, for liquid water, a threshold BE of 9.9 eV was found by Winter et al.,^46^ while 10.12(15) eV was found by Perry et al.^49^ The VBEs in our experiment are among the lowest reported for water clusters (see, e.g., refs (55, 56) and references therein for values by others), and settle between the reported bulk liquid values. Based on the here still resolved VBE shift between the two sizes, and the trend of results from various other experiments compiled by Gartmann et al.,^55^ we expect that the VBE of our (larger) pure water clusters is still up to a couple of hundred meV above the asymptotic bulk limit. This should be noted since comparably small differences in the solute VBEs between clusters and bulk solutions are considered below. The majority of the solute VBEs from liquid jet experiments that we compare our results with have been calibrated by setting the 1b_1_ VBE to the value of Winter et al.^46^ at 11.16 eV. Kurahashi et al.^48^ later suggested that the value should be refined to 11.31(4) eV after eliminating the effect of the streaming potential. As noted, the matter still remains under discussion,^52−54^ but the trend of 1b_1_ VBE of water clusters^55^ anyway seems to converge toward a similar energy range as the above-mentioned liquid jet values by Kurahashi et al.^48^ and Winter et al.^46^ The much lower temperature of the clusters may result in some discrepancy in the 1b_1_ VBE between the large cluster size limit and liquid jets. Under the assumptions that the 1b_1_ VBE decreases monotonously with increasing cluster size^55^ and increasing temperature,^46^ a slightly higher 1b_1_ VBE would be expected for neutral water clusters. When considering the size–energy relation of clusters probed in supersonic beams, it should be realized that the ionization probability of a cluster is proportional to the number of molecules in it, and therefore, the photoelectron spectra effectively represent a larger size distribution than the actual beam composition^55^ (depending also on the probe depth for sizes when the electron mean free path becomes comparable/shorter than the dimensions of the cluster). This also applies to considering the effectively probed salt concentration for the mixed salt–water clusters, which are discussed next.

### Pickup Process

**Figure 3:**
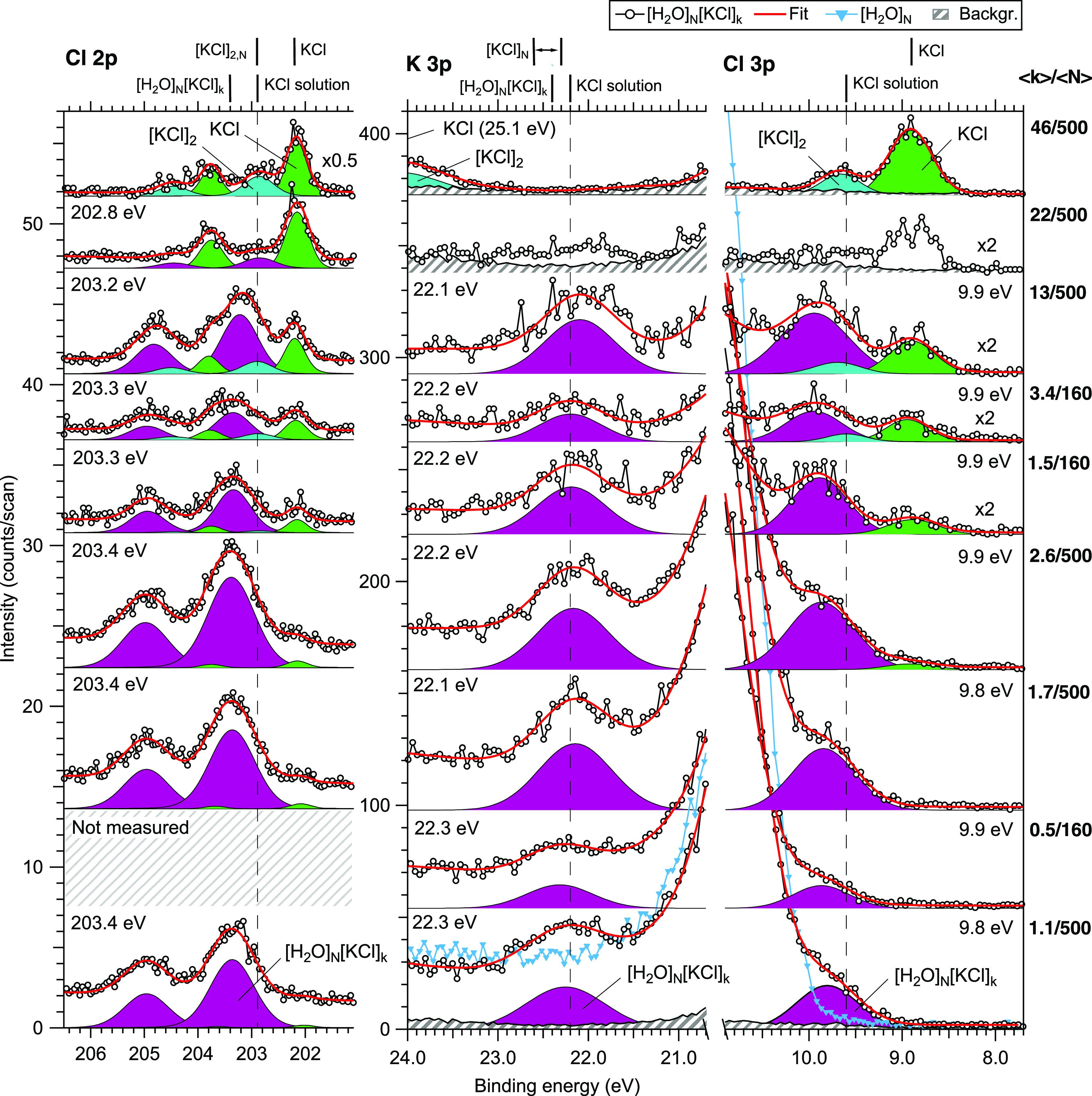
Photoelectron spectra in the Cl 2p (left), K 3p (center), and Cl 3p (right) regions. From bottom to top, the spectra are organized in order of increasing concentration, ⟨*k*⟩/⟨*N*⟩, where ⟨*k*⟩ denotes the (lower limit of) mean number of picked-up molecules and ⟨*N*⟩ is the mean initial cluster size (160_50_^250^ or 500_100_^1000^). Vertical offsets have been applied for clarity. The Cl 2p spectra were measured with a photon energy of 230 eV, while the K 3p and Cl 3p spectra were measured with 100 eV. Background (scaled independently) and pure water cluster spectra are also shown for comparison. VBEs of the fitted mixed cluster peaks are designated in the corner of each spectrum. For comparison, experimental VBEs from other reports are given on top of the graph for KCl,^14^ [KCl]_*N*_,^14^^14^ [H_2_O]_*N*_[KCl]_*M*_,^11^ and KCl solutions.^16,25^

Upon heating the pickup cell, signatures from KCl-doped water clusters gradually appeared in the photoelectron spectra, as seen in Figures 2 and 3. In the pickup cell, the salt evaporates mainly as neutral KCl monomers and to a lesser extent as [KCl]_2_ dimers, which are adsorbed on the initially pure water clusters. Distinct from the earlier experiments,^11,12^ a double skimmer configuration and a longer cluster flight path were used, which allowed extraction of the mixed cluster spectra (particularly the outermost valence) with the less overlapping signal from free KCl and [KCl]_2_ effusing from the pickup oven.^11,12^ The mean number of picked-up molecules ⟨*k*⟩ follows a Poisson distribution^57^ and scales with the density ρ ∼ *p*(*T*)/*T*_P_ of KCl, where *p*(*T*) is the vapor pressure^58^ and *T*_P_ is the temperature of KCl. More specifically, the given values represent the estimated lower limit of ⟨*k*⟩ and, accordingly, the true values should be somewhat higher. The estimation procedure of ⟨*k*⟩ is described in the Supporting Information.

The roles of low temperature, finite size, and limited relaxation time before the X-ray probe are to be considered when discussing the accommodation process of the salt to the water cluster. The initial cluster size is well above the requirements to favor charge separation with low concentrations.^6,59−61^ In bulk conditions, the first hydration shell of both K^+^ and Cl^–^ ions contains ∼6 water molecules and the ion–water distance is ∼0.3 nm to the first hydration shell and ∼0.5 nm to the second.^62^ The cluster size is thus sufficiently large to form multiple hydration layers around the ions, at least when the number of available water molecules is concerned. Localization of the ions to the cluster interior/surface is of interest. Recent studies suggest that in both clusters and bulk solutions, (heavy) halide anions are favorably positioned near the liquid–vapor interface, while alkali cations are more likely found below the immediate surface.^21,23,63−66^ The population of surface/interior sites likely differs between clusters and bulk solutions,^16^ considering the large surface-to-volume ratio, surface curvature,^67^ and low-temperature effects. Molecular dynamics (MD) simulations by Zhao et al.,^64^ Sun et al.,^68^ and Liu et al.^69^ on ion-containing water clusters suggest that Cl^–^ ions have a tendency to locate near to the cluster surface region but do not necessarily lie explicitly at the surface. In our experiment, one should however specifically consider the adsorption and solvation of an alkali halide molecule into a cold water cluster, and we are unaware of any simulations performed on directly analogous conditions. Charge separation is likely, despite the low internal temperature of the water clusters, as our (and earlier^11^) results also suggest. Alkali halides are known to readily dissolve also when adsorbed on ice films.^70−73^ However, if the cluster is frozen, one can argue whether a rigid water network will reduce the mobility of the salt and even prevent submergence of the ions.^73^ In similar experiments, various atomic and molecular species (and electrons) adsorbed on water clusters have been observed to remain on the surface.^42,74−76^

With alkali halides, a significant amount of energy is however brought into the system in the pickup process. We estimate that, in a single pickup event of KCl, the collision energy (internal energy of KCl + kinetic energy of the collision) is on the order of ∼500 meV, and that a further ∼2300 meV is released from charge separation (see the Supporting Information for calculation). Assuming that these energies are fully transferred to the internal energy of the cluster, the cluster temperature is raised by several tens of Kelvins, depending on the cluster size (rough estimates for the temperature change are provided in the Supporting Information). This means that if the water cluster is initially frozen, melting is potentially induced, especially when multiple pickup events occur. This may induce substantial reorganization of the cluster, e.g., migration of ions to the interior in the sub-nanosecond timescale^68^. Due to the simultaneously increasing rate of evaporative cooling, such high-temperature states would however be much shorter-lived than the approximately half a millisecond flight time of the clusters between pickup and the X-ray probe, given a cluster speed of ∼1000 m/s. Considering the evaporative cooling simulations on pure and ion-containing water clusters by Caleman and van der Spoel,^77,78^ we do not expect to be probing clusters at temperatures ≫200 K (depending also on the salt content). Thus, similar to the pure water clusters, the internal temperature of the mixed salt–water clusters should also be inevitably lower than accessible, e.g., in liquid jets.^16^ On the other hand, such low temperatures are typical for atmospheric particles, especially at high altitudes.

With an increasing number of picked-up molecules, evaporative cooling reduces the cluster size and therefore also effectively increases the concentration. The combined collision + solvation energies correspond to the binding enthalpy of ∼6 water molecules.^79^ Changes in the cluster size and concentration distribution are also subject to the fact that the pickup probability and diffusion losses (transverse velocity gain in pickup events) depend on the cluster size. With an increasing pickup temperature, the cluster intensity was observed to decrease and eventually vanish completely (Figure 2), which is attributed to the evaporation and diffusion effects. As this intensity decrease thus correlates with the amount of pickup, we have taken advantage of it in estimating ⟨*k*⟩, as described in the Supporting Information.

### Mixed KCl–Water Clusters

The acquired Cl 2p, K 3p, and Cl 3p regions are shown in Figure 3, measured in the *T*_P_ = 480–580 °C temperature range. The spectra on the right-side column are the same as shown in Figure 2, but here the Cl 3p region is magnified and shown with the peak fits. In the majority of the fits, the VBE of the Cl 3p level is found at ∼9.9 ± 0.1 eV, being ∼1 eV higher than the free KCl monomer at 8.9 ± 0.1 eV. We are unaware of other reported experimental Cl 3p VBEs in water clusters of this size regime. Markovich et al.^1^ have measured the VBE in the range 3.61–6.88 eV for smaller anionic Cl^–^[H_2_O]_0–7_ clusters. With few to few tens of water molecules in the cluster, the binding energies are highly sensitive to the precise number of water molecules and ions in the cluster, as well as to the cluster isomer and charge state.^1,5,6,12,63,80,81^ With hundreds of water molecules in the present experiment, this sensitivity appears to be notably suppressed. However, as it was already stated that the 1b_1_ VBE of the solvent has not explicitly converged to the asymptotic limit, there is reason to expect a slight deviation from bulk values also for the solute VBEs. Since in small clusters, the Cl 3p VBE gradually increases with an increasing number of water molecules,^1,81^ one might expect for the present results to settle below the bulk solution value. In contrast, in all spectra, the VBEs are higher than the 9.5^48^–9.6 eV,^16,25,38,82^ consistently reported from liquid jets. The ionization threshold is correspondingly higher, being 9.1 ± 0.1 eV at the lowest, while 8.7 eV has been reported from liquid jet solutions.^16^ The VBE of the Cl 3p level has gained considerable theoretical interest in recent years.^83−87^ Very comparable energies to our result were recently reported e.g., by Bouchafra et al.^85^ on Cl^–^ surrounded by 50 water molecules. Olleta et al.^81^ have calculated 8.51–9.90 eV for KCl(H_2_O)_0–6_ (some of their equilibrium structures are depicted in the abstract figure).

Photoelectron spectra of the anion core level, Cl 2p, are shown in the left-hand column of Figure 3. The spin–orbit splitting is ∼1.6 eV and, for convenience, we shall refer to VBEs of the 2p_3/2_ component. Previously, Partanen et al.^11^ found 203.4 ± 0.2 eV for NaCl and KCl in water clusters with ⟨*N*⟩ ∼ 150–400, but the effect of concentration was not investigated (the number of picked-up molecules was estimated to be <10). In our spectra, the VBEs range from 203.2 ± 0.1 to 203.4 ± 0.1 eV. The best agreement with that of Partanen et al.^11^ is found with low salt concentrations, as expected. Similar to the Cl 3p valence level, the Cl 2p VBEs are ∼1 eV higher than that of a free KCl molecule at 202.2 ± 0.1 eV, and also higher than the 202.89 eV microjet value reported by Pokapanich et al.^25^ for a 2 mol/kg (∼14 water molecules per ion) KCl solution.

For the cation, the K 3p region was acquired in the same scan range with the valence spectra, shown in the center column of Figure 3. Broader views of these spectra are included in the Supporting Information. The K 3p VBE in clusters is ∼3 eV lower than in the free KCl monomer, in contrast to the anion levels that were found in clusters at higher VBEs than the monomer. The opposite directions and magnitudes of the VBE shifts relate to the increasing and decreasing strengths of electrostatic interaction with the solvent in going from the initial state to the final state, as removal of an electron changes the charge state from +1 to +2 for a cation and from −1 to 0 for an anion.^11,13,82^ Previously, a K 3p VBE of 22.4 ± 0.1 eV was reported by Partanen et al.^11^ for KCl, KBr, and KI in water clusters. In our spectra, the fitted VBEs range from 22.1 ± 0.1 to 22.3 ± 0.1 eV. As for Cl 2p, the best agreement is found with the result of Partanen et al.^11^ in the lowest concentrations for K 3p as well. In liquid jets, a counterion-independent K 3p VBE of 22.2 eV has been reported,^16,25,82^ which is within the error bars of our spectra.

As such, better agreement in VBEs is found with liquid jets for the cation than for the anion, but as already noted the direct comparison of VBEs between clusters and bulk solutions is subject to a potential calibration offset. However, it seems that this can only partly explain the discrepancies, as agreement cannot be found for all three levels simultaneously by simply shifting the energy scale in any of our spectra. Most of the referred liquid jet results were calibrated with the 1b_1_ VBE set at 11.16 eV, and if we applied this same calibration procedure the agreement would improve for the anion levels but worsen for the cation.

### Structural Properties

As discussed above, the population of solvation sites likely differs between clusters and bulk solutions, although calculations do suggest that VBE shifts between surface/interior solvated ions should be rather subtle.^1,63,86,88,89^ We are unaware of any liquid jet studies where VBE differences between surface/interior sites would have been resolved, e.g., by depth profiling, whereas some studies on saturated solutions on substrates have reported large splitting of the anion core levels (the cation spectra showed only a single component).^65,66,90^ Various possible origins for this splitting were discussed by Tissot et al.^66^ For NaCl and RbCl solutions, the Cl 2p surface component was found ∼2 eV higher than the interior component,^66^ which is much larger than the here observed concentration-dependent shifts or VBE discrepancies to liquid jets, suggesting that we are not probing similar states.

Despite the relatively high resolution in the present experiment, no clear multipeak structures are resolved, which could allow a quantitative analysis of the population of qualitatively different chemical states such as surface/interior localized ions or solvent separated/shared/contact ion pairs (a clear two-peak structure was however observed previously for the Rb 3d core level^12^). This is in line with liquid jet observations,^16^ and does not, however, necessarily exclude the presence of these different structures in the clusters as a large number of variable solvent configurations may spread in energy with significant overlap so that they are indistinguishable. We emphasize that the spectra represent statistics from a broad range of equilibrium cluster structures and sizes, where the degree of disorder (which depends also on temperature) is reflected in the broad peak widths.^4,16,91,92^ The peak widths in clusters are similar to or slightly narrower than those in liquid jets for Cl 2p ∼0.7 to 1.0 eV (1 eV for the bulk solution^25^) and for K 3p ∼0.8 to 1.0 eV (1.4 ± 0.2 eV^16,82^). The same was found also for Br 3d in the RbBr experiment^12^ with widths between 0.55 and 0.80 eV (1.1/1.2 ± 0.10 eV^16^). For Cl 3p, we find ∼0.7 to 0.9 eV, while two rather different values have been reported from bulk solutions (0.6 ± 0.20^16^ and 0.9 ± 0.30^48^). If one assumes that, in clusters and bulk solutions, similar local (within the nearest hydration shells) solute–solvent structural moieties are probed, the differences in BEs and FWHM can be considered to reflect on the role of long-range electrostatic effects and the lower temperature of the clusters. It is however clear that the overall cluster structures can be different from perfect “snapshots” of bulk solutions due to their lower temperature, high surface area, and the fact that they are not confined in the solution, which allows additional freedom for the structural organization. Evidently, the cluster structures depend on the solvation dynamics during the pickup process, as discussed above.

### Concentration Effects

In an earlier experiment with another alkali halide, RbBr in water clusters, VBE shifts were observed with increasing pickup in the probed Rb 3d and Br 3d core levels, which were attributed to increasing ion pairing.^12^ The anion core level (Br 3d) was observed to shift to lower VBE and simultaneously the peak width was narrowed. Here, with KCl, the Cl 2p level behaves similarly. A particular −0.15 eV jump is observed in the ⟨*k*⟩ ∼ 13 spectrum, in which the water content in the clusters relative to salt is significantly lower as seen in Figure 2. The higher concentration spectra with ⟨*k*⟩ ∼ 22 and 46 are found at still lower VBEs, but the broad peaks likely contain a significant amount of [KCl]_2_ as well, and the cluster contribution (and VBE) cannot be reliably assessed. The signal from water in these clusters is (almost) negligible. In the highest temperature spectra (⟨*k*⟩ ∼ 46), the observed features in all three levels seem to originate from free KCl and [KCl]_2_ only. It can be considered that when concentration increases, the Cl 2p VBE shifts slightly closer to that reported for both anhydrous ([KCl]_*N*_^11,14^) and hydrous (liquid KCl solution^25^) multi-ionic systems, which coincide at ∼202.9 eV. Note that the number of picked-up molecules is not, in principle, limited by solubility. For the cation core level Rb 3d (VBE ∼ 115 eV), a second feature at ∼1 eV higher VBE was observed with increasing concentration of RbBr,^12^ where the lower VBE feature was attributed to fully solvated ions and the higher VBE feature to solvent shared and contact ion pairs. Here, we do not resolve similar splitting for the K 3p level, which may reflect that K 3p is a valence orbital and does not express comparably large VBE shifts. Some variation is found in the average VBE and FWHM of K 3p over the probed concentration range, but not in an entirely monotonous manner. We refrain from fitting the K 3p and Cl 3p spectra with ⟨*k*⟩ ∼ 22 due to low statistics, but we note that in both levels signal is still observed at energies comparable to the lower temperature spectra, albeit that there is an almost negligible signal from water in the clusters.

The effect of concentration has been addressed also for bulk solutions in liquid jets but negligible or only very subtle VBE shifts have been reported.^16,51,82,93^ We are unaware of any reported changes in Cl 2p, K 3p, and Cl 3p VBEs with changing alkali halide concentration in liquid jets, besides that two rather different VBEs have been observed for Cl 2p in separate experiments (202.89 eV for 2 mol/kg KCl^25^ and 202.1 eV for 3 mol/kg LiCl^91^), which Pokapanich et al. considered to possibly indicate the dependence of the VBE on counter ion or concentration. Recently, Pohl et al.^51^ studied NaI solutions over a broad concentration range of 0.5–8.0 M (∼56 to 3 water molecules per ion). They reported that the VBE of the I 4d level decreased up to 0.15 ± 0.06 eV and the 5p valence level up to 0.11 ± 0.06 eV, while no shift was observed for the Na 2p level of the cation (referencing the energies to the 1b_1_ level of the solution). They speculated that at higher concentrations, charge donation from the anion to the solvent is affected as the water network becomes increasingly disrupted. The slightly decreasing VBE of the anion core level (I 4d) is qualitatively similar to that found here for Cl 2p and previously for Br 3d^12^ in water clusters. There are however also cluster-specific effects that must be considered in assessing the origin of the observed concentration dependence of VBEs and FWHMs in clusters. As discussed above, besides the increasing salt concentration, the cluster size and temperature are also varied with an increasing number of adsorbed salt molecules. In this sense, the observations are not entirely comparable to the bulk solution case.

Since the clusters are generated essentially by in-vacuum condensation of neutral water and salt molecules, they can be considered to be electrically neutral and contain an equal number of cations and anions. This assumption may break down if charge evaporation from the pickup oven or from the clusters occurs during the pickup process, which could also lead to VBE shifts, although we expect this effect to be minor at least when the number of picked-up molecules is small. We emphasize that the results are however not subject to structural changes subsequent to photoionization due to the instantaneous nature of the ionization process and that no permanent radiation damage can play a role due to the constantly renewable particle beam.

Finally, the effect of the solute on the solvent electronic levels is also of interest, particularly to the VBE of 1b_1_. In Figure 2, the cluster 1b_1_ peak is seen to decrease in intensity with increasing salt content, but also its shape is modified and weighted to higher VBEs. Similar behavior was observed also in the RbBr experiment.^12^ This implies that shifting of the solvent VBEs may occur due to the effect of the ions. We note that this would contrast the conclusion of Pohl et al.^51^ that, in a bulk solution, the electrolyte (NaI) has a minimal effect on the 1b_1_ VBE, justifying its use as a calibration reference. However, as discussed for the solute spectra, cluster-specific effects can play a role here as well. Both pure and mixed clusters are probed simultaneously with varying relative intensities, which are inseparable from the solvent spectra. Besides the salt concentration, size distributions of the pure and mixed cluster fractions are modified since the pickup probability as well as evaporation and diffusion effects depend on the cluster size and the number of picked-up molecules (see also, e.g., ref (94)), which can also be responsible for the observed BE shift. Further investigation would be needed for clarification.

## Conclusions

Nanosolvation of KCl in gas-phase water clusters has been investigated with core and valence level photoelectron spectroscopy. The results indicate that with hydration states of a few hundred water molecules, the electronic structure of the solute no longer shows high sensitivity to cluster size, as reflected in the VBEs of the probed Cl 2p, Cl 3p, and K 3p levels. Yet, the observed VBEs and FWHM do not fully agree with those observed near the liquid–vapor interface of bulk solutions. In particular, the VBE of the Cl 3p valence level at 9.9 ± 0.1 eV, as well as the Cl 2p core level, do not settle between that of the free KCl monomer (8.9 ± 0.1 eV) and liquid jet solutions (9.6 ± 0.07 eV^16^). Further investigations are needed to clarify the role of unique structural properties, low-temperature effects, calibration differences, and the finite extent of long-range electrostatic ion–ion and ion–solvent interactions in the nanoaqueous environment. In future experiments, we expect that the structural properties of the clusters will be further elucidated by the combination of electron spectroscopy with other techniques such as X-ray absorption spectroscopy,^95,96^ mass spectroscopy,^44,55,75^ and multiparticle coincidence experiments.^97^ The reported experimental VBEs offer relevant benchmarks for theoretical works.

## Supporting Information Available

Details of the clustering conditions and data collection; peak fitting procedures; pickup estimation; estimation of collision and solvation energies; and broader views of the photoelectron spectra (PDF)
