# Supplementary material for: Core and Valence Level Photoelectron Spectroscopy of Nanosolvated KCl
Source: J Phys Chem A. 2021 May 26;125(22):4750–9. doi: 10.1021/acs.jpca.1c01539 (PMC8279652; doi:10.1021/acs.jpca.1c01539)
Supplement: Supplementary file 1 — jp1c01539_si_002.pdf [file jp1c01539_si_002.pdf]

Supporting Information: Core and Valence Level Photoelectron Spectroscopy of Nanosolvated KCl

Eetu Pelimanni,[*,†] Lauri Hautala,[†] Andreas Hans,[‡,†] Antti Kivimäki,[†,¶] Mati Kook,[§] Catmarna Küstner-Wetekam,[‡] Lutz Marder,[‡] Minna Patanen,[†] and Marko Huttula[†]

†*Nano and Molecular Systems Research Unit, Faculty of Science, University of Oulu, Box 3000, FI-90014, Finland*

‡*Universität Kassel, Institut für Physik und CINSaT, Heinrich-Plett-Straße 40, 34132 Kassel, Germany*

¶*MAX IV Laboratory, Lund University, P.O. Box 118, SE-22100 Lund, Sweden*

§*Institute of Physics, University of Tartu, W. Ostwaldi 1, EE-50411 Tartu, Estonia*

E-mail: eetu.pelimanni@oulu.fi

Details of the spectra

Table S1: Table of clustering conditions and number of recorded scans. T_P(°C) is given to 5 °C accuracy.

	T_L(°C)	T_N(°C)	P(mbar)	$\langle N \rangle$	T_P(°C)	$\langle k \rangle$	hv=100 eV #scans	hv=230 eV #scans
H_2O	80	100	500	160	-	0	18	-
H_2O	80	100	500	160	485	0.5	4	-
H_2O	80	100	500	160	510	1.5	3	15
H_2O	80	100	500	160	530	3.4	3	15
H_2O+Ar	80	100	1000	500	-	0	5	-
H_2O+Ar	80	100	1000	500	485	1.1	7	40
H_2O+Ar	80	100	1000	500	495	1.7	3	40
H_2O+Ar	80	100	1000	500	505	2.6	3	35
H_2O+Ar	80	100	1000	500	545	13	2	29
H_2O+Ar	80	100	1000	500	560	22	3	20
H_2O+Ar	80	100	1000	500	580	46	5	5

Peak analysis

The $1b_1$ VBE of water clusters was fitted with a single symmetric Gaussian. Here the background reference spectrum was fitted first with a set of peaks describing the vibrational fine structure of water[1] and the minor contribution from vacuum impurities. These curve shapes were then used to account for their contribution in the cluster spectra. The $3a_1$ peak of condensed water is considered split and typically described with two peaks,[2] but as here we are mainly interested in the $1b_1$ feature, a single peak was used for simplicity to account for the partial overlap with $1b_1$. Additional necessary constraints were applied to relative positions and ratios of the peaks for the fitting algorithm to produce physically meaningful results.

The Cl 3p and K 3p features were fitted with symmetric Gaussian profiles. Single peak fits were used for simplicity and they mostly reproduce the observed features well. The pure water cluster spectra were first fitted separately and this result was then used as a starting point of the mixed spectra fits for the low concentrations.

For Cl 2p, the peak intensity ratio between the $2p_{1/2}$ and $2p_{3/2}$ components was fixed to the statistical state degeneracy 1:2. The spin-orbit splitting of the monomer peaks and the cluster peaks was found to be ∼1.6 eV, similar to e.g. in refs. 3,4, and this value was fixed for all (monomer, dimer and cluster) components to improve the fit quality and consistency. Lorentzian broadening was fixed to 100 meV for all Cl 2p peaks to account for lifetime broadening.[5] The Gaussian widths were forced to be the same for both spin-orbit components. The energy positions, intensities and Gaussian widths were otherwise left as free parameters in the fits.

The monomer and dimer peaks overlapping with the mixed cluster peaks were accounted for when necessary (with some additional constraints when their countrate was low). As a side result, their VBEs can be extracted from the highest temperature spectrum in which we assume a negligible contribution from clusters. The VBEs of KCl and $[KCl]_2$ are 8.9±0.1 eV & 9.7±0.1 eV for Cl 3p, 25.1±0.1 eV & 24.1±0.1 eV for K 3p and 202.2±0.1 & 202.8±0.2 for Cl $2p_{3/2}$, respectively.

Pick up estimation

The probability for a water cluster to pick up k salt molecules follows the Poisson distribution

$$P(k) = \frac{\langle k \rangle^k}{k!} e^{-\langle k \rangle}. \tag{1}$$

The expectation value $\langle k \rangle$, i.e. the mean number of picked up molecules, is

$$\langle k \rangle = \rho \cdot \sigma \cdot L = \frac{p(T)}{k_B T_P} \cdot \sigma \cdot L, \tag{2}$$

where σ is the pick-up cross section, L is the length of the pick up cell, $\rho = p/(k_B T_P)$ is the number density of KCl, p is the vapor pressure of KCl and T_P is the temperature of KCl.[6] It turns out that given the uncertainties in determining the effective σ,[7,8] L and ρ, direct

application of this equation is not justified. We therefore estimate $\langle k \rangle$ from the observed fall of the water cluster signal intensity, which occurs due to evaporative cooling of the clusters and due to diffusion losses, and therefore correlates with the true amount of occurring pick up. The relative peak height (counts at peak maximum per scan) of the $1b_1$ valence band for the smaller clusters (due to its higher sensitivity to both diffusion and evaporation) is plotted as a function of pick up temperature in figure S1 (red circles).

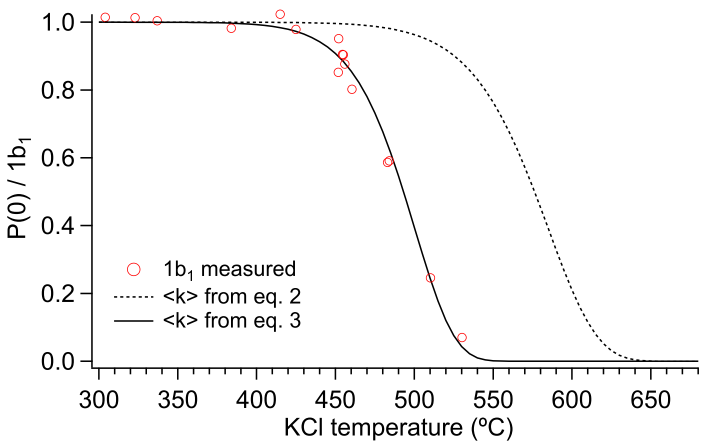

Figure S1: Temperature dependent $1b_1$ intensity drop of the smaller cluster size together with calculated P(0) using $\langle k \rangle$ from equations 2 (theor.) and 3 (f corrected).

A maximum possible intensity drop occurs if it is assumed that none of the clusters with $k \geq 1$ are observed. In this case, the cluster intensity should follow P(0) (eq. 1). The calculated P(0) according to eq. 1 is shown by the dashed line, using the length of the pick up cell L=12.5 mm and a geometrical cross section of the water cluster $\sigma = 358\,\text{Å}^2$ (calculated as in ref.[9] and refs. therein). The calculation is clearly an underestimation. There are several possible reasons for this, including slight inaccuracy in the temperature measurement and L and σ being effectively larger.[7] Therefore, we simplify eq. 2 to

$$\langle k \rangle = \frac{p(T)}{T_P} \cdot f. \tag{3}$$

Here the scaling of $\langle k \rangle$ with the changing temperature is retained, and f is a constant that accounts for the effective σ and L (and $1/k_B$) as well as uncertainties of the measured T_P

and correspondingly calculated[10] $p(T)$. By selecting f to match with the observations (solid black line in fig. S1), a lower limit of true $\langle k \rangle$ is obtained, being ~25 times larger than calculated according to eq. 2. This approach bypasses the need for accurate knowledge on the different parameters in eq. 2. Yet, acknowledging the crudeness of the estimation, the discussion has been accordingly kept at a qualitative level. To account for the increasing pick up probability with increasing cluster size, eq. 3 is further multiplied with $\sigma_L/\sigma_S = 2.1$, where σ_L and σ_S are the geometrical cross sections of the clusters calculated for $N = 500$ & $N = 160$, respectively.

Temperature change of the clusters

Crude estimations for the internal energy changes involved in the pick up process are presented. Since the cluster beam contains a distribution of cluster sizes, the estimated temperature change of a water cluster upon a pick up event and upon evaporation of water are plotted as a function of cluster size N in figure S2. The curves assume that the energy change is fully transformed to internal heat of the cluster. The temperature change of a water cluster is calculated as

$$\Delta T \sim \frac{\Delta H}{(C_m N)}, \tag{4}$$

where ΔH is the internal energy change, C_m is the heat capacity per molecule and N is the number of water molecules. In general, C_m depends on cluster size and temperature,[11] but for simplicity we use a representative value of 0.25 meV/K/molecule. The binding enthalpy of an evaporating water molecule likewise depends on cluster size and composition, and we use a representative value of 500 meV for the plot, which is also between the values in bulk liquid water and bulk water ice.[12]

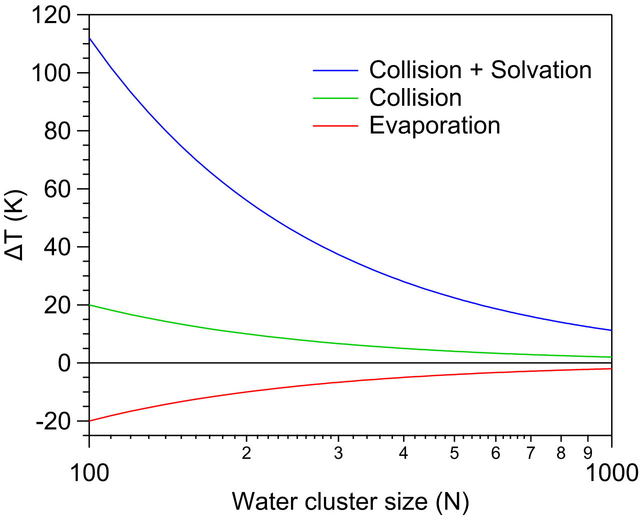

Figure S2: Temperature change of a water cluster in a single pick up event accounting for the collision or the combined collision + solvation energies. The temperature change upon evaporation of a single water molecule is also shown.

The mean collision energy between a water cluster and a free KCl molecule is[13,14]

$$\langle E_{col}\rangle = 2k_BT + \frac{3}{2}k_BT + \frac{1}{2}mv^2, \qquad (5)$$

where T is the temperature of the salt, m is the mass of KCl and v is the cluster beam velocity. The first term accounts for the internal energy (vibrational + rotational) of KCl and the latter two account for the kinetic energy. The kinetic part assumes a Maxwellian velocity distribution for KCl and a sharply peaked velocity for the clusters. The cluster velocity was calculated similarly as described e.g. in ref. 9. Eq. 5 results to ∼500–800 meV, where variation is due to slightly different cluster beam velocities and the temperature range of the salt. The curves in fig. S2 are plotted using a value of 500 meV.

The energy release in the solvation of a single KCl molecule from the gas phase is calculated as a sum of enthalpy of dissociation of

$KCl(g) \rightarrow Cl^-(g) + K^+(g)$ $\quad$ ($481.2\,kJ/mol$[15])

and subsequent hydration enthalpies

$K^+(g) \rightarrow K^+(aq)$ $\quad$ ($-322\,kJ/mol$[16])

$$Cl^-(g) \rightarrow Cl^-(aq) \qquad (-381\,kJ/mol^{16})$$

This adds to -222 kJ/mol ∼ -2.3 eV.

Photoelectron spectra measured with hv=100 eV

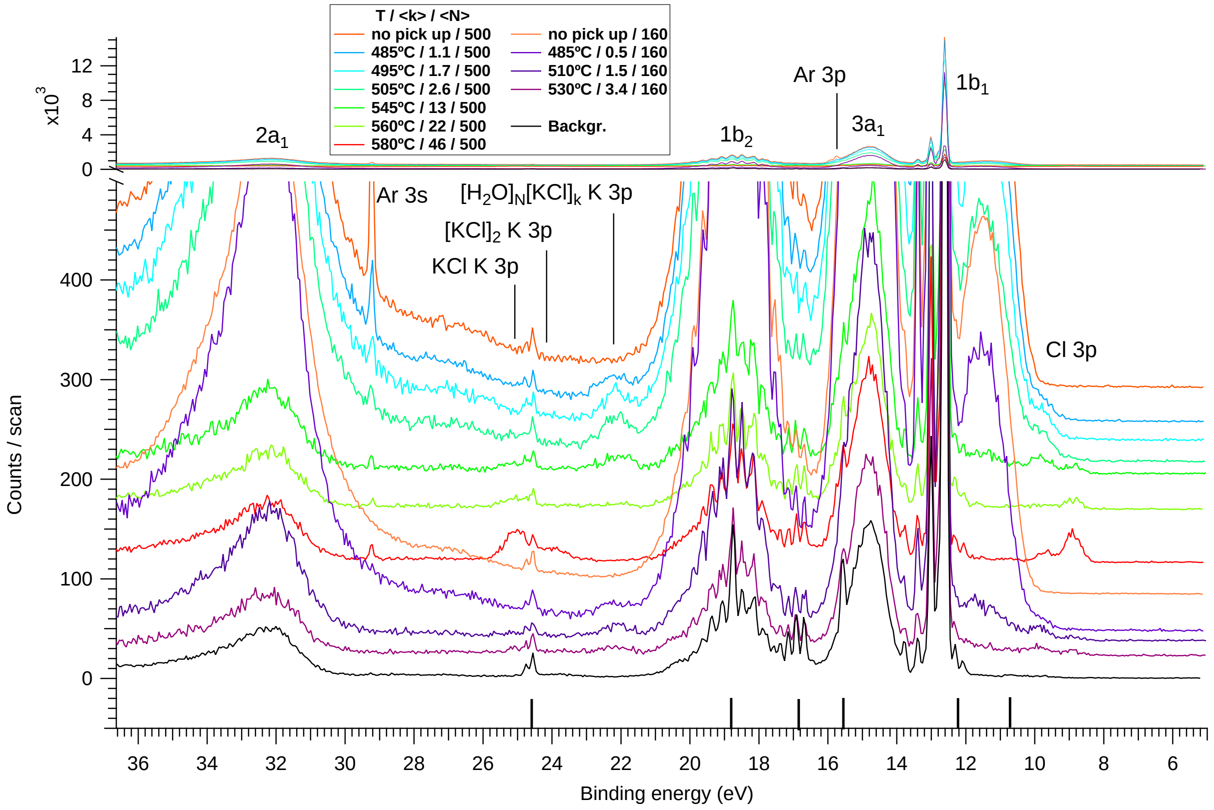

Figure S3: A broader view of the low BE spectra. A few vacuum impurity features are indicated with the thick vertical lines at the bottom.

References

(1) Karlsson, L.; Mattsson, L.; Jadrny, R.; Albridge, R. G.; Pinchas, S.; Bergmark, T.; Siegbahn, K. Isotopic and Vibronic Coupling Effects in the Valence Electron Spectra of $H_2{}^{16}O$, $H_2{}^{18}O$, and $D_2{}^{16}O$. *The Journal of Chemical Physics* **1975**, *62*, 4745–4752.

(2) Pohl, M. N.; Muchová, E.; Seidel, R.; Ali, H.; Sršeň, Š.; Wilkinson, I.; Winter, B.;

Slavíček, P. Do Water's Electrons Care About Electrolytes? *Chem. Sci.* **2019**, *10*, 848–865.

(3) Patanen, M.; Bancroft, G. M.; Aksela, S.; Aksela, H. Direct Experimental Determination of the K 2p and Cl 2p Core-level Binding Energy Shifts Between Molecular and Solid KCl: Line Broadening Effects. *Phys. Rev. B* **2012**, *85*, 125419.

(4) Pokapanich, W.; Bergersen, H.; Bradeanu, I. L.; Marinho, R. R. T.; Lindblad, A.; Legendre, S.; Rosso, A.; Svensson, S.; , O. B.; Tchaplyguine, M. et al. Auger Electron Spectroscopy as a Probe of the Solution of Aqueous Ions. *Journal of the American Chemical Society* **2009**, *131*, 7264–7271.

(5) Travnikova, O.; Fink, R. F.; Kivimäki, A.; Céolin, D.; Bao, Z.; Piancastelli, M. N. Disentangling the Complex Line Profiles in the Cl 2p Photoelectron Spectra of Cl_2. *Chemical Physics Letters* **2006**, *426*, 452 – 458.

(6) Behrens, M.; Fröchtenicht, R.; Hartmann, M.; Siebers, J.-G.; Buck, U.; Hagemeister, F. C. Vibrational Spectroscopy of Methanol and Acetonitrile Clusters in Cold Helium Droplets. *The Journal of Chemical Physics* **1999**, *111*, 2436–2443.

(7) Lengyel, J.; Kočišek, J.; Poterya, V.; Pysanenko, A.; Svrčková, P.; Fárník, M.; Zaouris, D. K.; Fedor, J. Uptake of Atmospheric Molecules by Ice Nanoparticles: Pickup Cross Sections. *The Journal of Chemical Physics* **2012**, *137*, 034304.

(8) Lengyel, J.; Pysanenko, A.; Poterya, V.; Slavíček, P.; Fárník, M.; Kočišek, J.; Fedor, J. Irregular Shapes of Water Clusters Generated in Supersonic Expansions. *Phys. Rev. Lett.* **2014**, *112*, 113401.

(9) Hautala, L.; Jänkälä, K.; Mikkelä, M.-H.; Turunen, P.; Prisle, N. L.; Patanen, M.; Tchaplyguine, M.; Huttula, M. Probing RbBr Solvation in Freestanding sub-2 nm Water Clusters. *Phys. Chem. Chem. Phys.* **2017**, *19*, 25158–25167.

(10) Bradley, R. S.; Volans, P.; Whytlaw-Gray, R. Rates of Evaporation VI. The Vapour Pressure and Rate of Evaporation of Potassium Chloride. *Proceedings of the Royal Society of London. Series A. Mathematical and Physical Sciences* **1953**, *217*, 508–523.

(11) Hock, C.; Schmidt, M.; Kuhnen, R.; Bartels, C.; Ma, L.; Haberland, H.; v.Issendorff, B. Calorimetric Observation of the Melting of Free Water Nanoparticles at Cryogenic Temperatures. *Phys. Rev. Lett.* **2009**, *103*, 073401.

(12) Heiles, S.; Cooper, R. J.; DiTucci, M. J.; Williams, E. R. Sequential Water Molecule Binding Enthalpies for Aqueous Nanodrops Containing a Mono-, Di- or Trivalent Ion and Between 20 and 500 Water Molecules. *Chem. Sci.* **2017**, *8*, 2973–2982.

(13) Lewerenz, M.; Schilling, B.; Toennies, J. P. Successive Capture and Coagulation of Atoms and Molecules to Small Clusters in Large Liquid Helium Clusters. *The Journal of Chemical Physics* **1995**, *102*, 8191–8207.

(14) Hautala, L. Synchrotron Radiation Based Characterization of Structural Evolution of Alkali Halide Clusters. Ph.D. thesis, University of Oulu, 2017.

(15) Brewer, L.; Brackett, E. The Dissociation Energies of Gaseous Alkali Halides. *Chemical Reviews* **1961**, *61*, 425–432.

(16) Smith, D. W. Ionic Hydration Enthalpies. *Journal of Chemical Education* **1977**, *54*, 540.
